# Investigation of *Plantago ovata* Husk as Pharmaceutical Excipient for Solid Dosage Form (Orodispersible Tablets)

**DOI:** 10.1155/2021/5538075

**Published:** 2021-06-14

**Authors:** Sarmad Abbas, Mehrin Sherazi, Amjad Khan, Hamad S. Alyami, Muhammad Latif, Zia-Ur-Rahman Qureshi, Majeedullah, Muhammad Hassham Hassan Bin Asad

**Affiliations:** ^1^Department of Pharmacy, Abasyn University, Peshawar, Pakistan; ^2^Department of Pharmacy, Kohat University of Science and Technology (KUST), Kohat, Pakistan; ^3^Department of Pharmaceutics, Najran University, Najran, Saudi Arabia; ^4^Department of Zoology, Division of Science and Technology, University of Education Lahore (Multan Campus), Pakistan; ^5^Department of Pharmacy, SBK Women University, Quetta, Balochistan, Pakistan; ^6^Department of Pharmacy, COMSATS University Islamabad (Abbottabad Campus), 22060, Pakistan; ^7^Institute of Fundamental Medicine and Biology, Department of Genetics, Kazan Federal University, Kazan, Russia

## Abstract

The objective of the study was to investigate the suitability of the *Plantago ovata* (PO) husk as a pharmaceutical excipient. Various phytoconstituents of the husk were determined according to the standard test procedures. The *Plantago ovata* husk was evaluated for various pharmaceutical parameters related to flow, swelling index, and compressibility index. Orodispersible tablets (ODTs) were prepared, containing different concentrations (2.5, 3, 5, 7.5, 10, and 15% *w*/*w*) of the *Plantago ovata* husk. Before compression, all the formulations were evaluated for their flow. Compressed ODTs were evaluated for physical characteristics (physical appearance, weight and weight variation, thickness, and moisture content), mechanical strength (crushing strength, specific crushing strength, tensile strength, and friability), disintegration behavior (disintegration time and oral disintegration time), drug content, and in vitro drug release. Phytochemical evaluation of the *Plantago ovata* husk confirmed the presence of various phytoconstituents like alkaloids, tannins, glycosides, saponins, flavonoids, and phenols. SEM photograph of the *Plantago ovata* husk showed that it has a fibrous structure, with a porous and rough surface. The *Plantago ovata* husk had a high swelling index (380%) which decreased by pulverization (310%). Precompression evaluation of the powder blend for all the formulations of ODTs showed good flow properties, indicating that the *Plantago ovata* husk improved the rheological characteristics of the powder blend. Compressed ODTs had good mechanical strength, and their friability was within the official limits (<1%). Best disintegration was observed with formulation F-6 containing 10% *w*/*w* of the *Plantago ovata* husk. It is concluded that the *Plantago ovata* husk can be used as a disintegrant in the formulation of ODTs.

## 1. Introduction

*Plantago ovata* (PO) belongs to the genus *Plantago*, and with regard to its 200 known species, 10 species are found in the subcontinent. It is cultivated during November to April [1]. It is an herb and is 30–45 cm, with 7.5–20 cm long and 0.6 cm broad leaves [2]. It has 1.2–4 cm spikes which are 0.5 cm broad and bearing 45–69 flowers [3]. Fruits are ellipsoid capsules 8 mm long, glabrous, and obtuse, and seeds are ovoid-oblong, smooth, boat shaped, and rosy-white convex on one side and concave on another side. The husk is obtained by milling the seed, with a total recovery of 25–26% [2]. The husk consists of fibrous insoluble fibers (34%) and soluble fibers (66%). Its mucilage contains up to 85% of different carbohydrates like arabinose, xylose, and galacturonic acid [4]; 0.94% proteins (albumin, globulin, and prolamin); and 4.07% ash [5]. Traditionally, it is used as an antidiarrheal, laxative [6], antidiabetic, and antihyperlipidemic [7]. The husks of the PO Forsk seed are being used as a dietary fiber supplement to regulate large bowel functions, and in recent times, it has shown prominent blood cholesterol-lowering effects. Some of the pharmaceutical applications of PO have been reported. The husk shows negative thixotropy on dispersion in water; however, if heated to 60°C, it is transformed to a thixotropic gel with bulges and spurs. The mucilage has emulsification properties [8] and is a good suspension agent [9]. The 2% *w*/*v* mucilage (seed-husk powder) in cool water matches well, while 1.5% *w*/*v* mucilage in warm water is better than 10% *w*/*v* starch mucilage in terms of binding properties [10]. It is comparable to methyl cellulose and is much better than both sodium alginate and sodium carboxymethyl cellulose in suspending properties [9]. The carboxymethyl derivative of the PO husk is a fibrous mass, lighter in odor, and mucilaginous in taste. It swells well in water, making gummy mucilage of pH 6.7 and swelling factor 90. It is better than the integral husk in viscosity [11], spreadability, film creating features, and smooth texture [12]. The husk hazy aqueous extract is an exceptional thickening agent. The husk of PO seeds has good gelling and gliding properties. Furthermore, it absorbs water and swells to a larger extent. It is bulky in nature, with a low density, and is expected to increase flow characteristics of other powder materials. These characteristics of the PO husk make the basis for its proper investigation as a pharmaceutical excipient for solid dosage form (tablet).

A number of disintegrants are available with varying degrees of disintegration action. The main problem with available disintegrants is their effect on the mechanical strength of tablets [13, 14]. A higher concentration of disintegrants makes tablets prone to friability, so they should be used in lower recommended concentrations. Furthermore, higher concentration in orodispersible tablets (ODTs) can cause grittiness in the oral cavity [15]. There is a need for cost effective and safe disintegrants which can be used in larger quantities, without compromising the mechanical strength of tablets. Natural products are preferred due to their nontoxic, nonirritant, cost effectiveness, and local accessibility. In this study, domperidone was used as the model drug in the preparation of ODTs for determination of the suitability of the PO husk as a disintegrant. Domperidone is an antidopaminergic drug, used for the treatment of gastroesophageal reflux disorder (GERD), vomiting, and delayed gastric emptying [16]. It has poor water solubility, is a tasteless drug, and has a dose strength of 10 mg/tablet.

Materials of natural origin have been used in pharmaceutical preparations at various stages because of their safe nature, easy availability, and cost effectiveness. Our main objective was to investigate suitability of the PO husk as a pharmaceutical excipient for solid dosage form. In the present study, physicochemical characteristics of the PO husk were determined, and various formulations of ODTs of domperidone were developed, using the PO husk as the disintegrant. The obtained results were compared with commonly used pharmaceutical ingredients.

## 2. Material and Methods

### 2.1. Material

Domperidone (Ningbo Sansheng Pharmaceutical Company, China; purity 99.83% with respect to BP standard) was gifted by Nenza Pharmaceuticals, Peshawar, Pakistan. Other materials like the PO husk (Muhammad Hashmi Husks, Karachi, Pakistan), sodium starch glycolate (Primojel; CHP Carbohydrates, Pirna, Germany), magnesium stearate (Coin Powder International Company Ltd., Taiwan), Tablettose-80 (BDH Chemical Limited, Poole, England), microcrystalline cellulose (Dr. Reddy's Laboratories, India), and flavour (Fab Flavours and Fragrances Pvt. Ltd., India) were acquired locally in Peshawar, Pakistan. All these ingredients were of pharmaceutical quality and were utilized without any processing.

### 2.2. Instruments and Equipment

Instruments used for evaluation of physicochemical characteristics of the PO husk and preparation of ODTs included sieve shaker (Endicott Ltd., England), laboratory-scale double cone mixer (Morgan Machinery Ltd., Pakistan), rotary tablet compression machine ZP-19 (STC, China), rotary granulator (STC, China), digital balance (LIBROR AEG-120, Shimadzu, Japan), tablet disintegration apparatus (Pharma Test, Germany), dissolution testing apparatus (Pharma Test, Germany), digital tablet hardness and thickness tester (Pharma Test, Germany), double drum friabilator (Faisal Engineering, Pakistan), and UV-visible spectrophotometer (Shimadzu, Japan).

### 2.3. Pharmaceutical Evaluation of PO Husk

#### 2.3.1. Density

The husk bulk density was calculated as per the official USP method using a graduated cylinder [17]. In the graduated cylinder, the volume of the weighed quantity of powder was measured, and density was estimated by the equation *D* = *m*/*v* (equation (1)), where *D* is the density of the powder (g/mL), *m* is the weight of the powder, and *v* is the volume of the powder (mL). Tapped density of the PO husk was obtained by the USP method, i.e., by the tapping of powder in a graduated cylinder and noting the volume reduction [17]. Tapping was done manually. The final volume of the husk was taken as the tapped volume and was used for the calculation of the tapped density. All measurements were triplicated and the values presented as mean ± standard deviation (*n* = 3).

#### 2.3.2. Carr's Index

Carr's index was estimated based on the tapped and bulk densities of the PO husk [18], by the following equation: C.I = (Dc − Da/Dc) × 100  (equation (2)), where *C*.*I* is Carr's index of the powder (%), *Dc* is the tapped density of the powder (g/mL), and *Da* is the bulk density of the powder (g/mL).

#### 2.3.3. Hausner Ratio

The Hausner ratio was also assessed from the bulk and tapped density values of the PO husk [18] by the following equation: Hr = Dc/Da (equation (3)), where *Hr* is the Hausner ratio of the powder, *Dc* is the powder tapped density (g/mL), and *Da* is the powder bulk density (g/mL).

#### 2.3.4. Angle of Repose (AOR)

AOR was obtained as per the USP funnel method [18]. The PO husk was allowed to flow from a glass funnel fitted at a specified height, and AOR was calculated on the basis of the height and radius of the heap; ∝ = tan^−1^(*H*/*r*) (equation (4)), where *α* is the AOR of the powder (°), *H* is the cone height shaped by the powder (cm), and *r* is the cone base radius shaped by the powder (cm).

#### 2.3.5. Swelling Index

The swelling index of the PO husk was determined according to USP [18]. The weighed amount of the husk was taken in a graduated cylinder and its volume (*V*_1_) was measured. Sufficient quantity of purified water was added to the graduated cylinder containing the PO husk and kept undisturbed for 3 h. Volume of the swollen PO husk was noted (*V*_2_), and the swelling index was calculated by the following equation: Swelling index = (*V*_2_ − *V*_1_)/*V*_1_ × 100 (equation (5)), where *V*_1_ is the volume occupied by the dry husk and *V*_2_ is the volume of the swollen husk. The swelling index was determined in triplicate, and the mean and standard deviation were calculated.

#### 2.3.6. Interparticle Porosity

Bulk and tapped density values were used for estimation of the interparticle porosity [18] by the following equation: Ie = (Dc − Da)/Dc × Da (equation (6)), where *Ie* is the interparticle porosity, *Dc* is the tapped density (g/mL), and *Da* is the bulk density (g/mL).

#### 2.3.7. Loss on Drying (LOD)

LOD was obtained gravimetrically according to USP [18], using a halogen moisture analyzer. Approximately 1 g of powder was taken on an analyzer pan and heated for a stated time (5 min) at 100°C, and the percentage weight loss was calculated. LOD experiments were triplicated, and values presented as *mean* ± *SD* (*n* = 3).

#### 2.3.8. Compressibility

Compressibility of the PO husk was assessed on the basis of the cohesion index. The cohesion index is the crushing strength of the powder compressed in an eccentric press, under maximum pressure without capping and lamination [19]. The mean crushing strength was calculated for ten compactions (*n* = 10), indicating cohesion index of the PO husk.

### 2.4. Phytochemical Evaluation of PO Husk

The PO husk was studied for the presence of various classes of compounds, according to the standard protocols as follows.

#### 2.4.1. Test for Carbohydrates

The PO husk was soaked in purified water (5 mL) for 5 min and filtered. Alcoholic *α*-naphthol solution (3 drops) was added to the filtrate and taken in a test tube, and the junction was observed. The presence of carbohydrates was confirmed by the appearance of a violet ring at the junction.

#### 2.4.2. Test for Alkaloids

The presence of alkaloids in the PO husk was confirmed by Mayer's test. The PO husk (2.5 g) was extracted with methanol and HCl (2 N) was added. The mixture was heated at 40 ± 3°*C*, cooled, and filtered. This filtrate was reacted with Mayer's reagent (potassium mercuric iodide), and the precipitate growth was checked. The appearance of yellow colored precipitate confirmed the presence of alkaloids.

#### 2.4.3. Test for Saponins

The PO husk was soaked in boiling water in a test tube. After a specified time, the mixture was cooled and vigorously shaken till froth formation. Afterward, the test tube was placed on a stand for ~15 min, and the results were noted. Strongly positive (+++) meant more than 5 cm froth, (++) meant more than 2 cm froth, (+) meant <1 cm froth, and (-) represented no froth.

#### 2.4.4. Test for Phenols

The PO husk was soaked in water and filtered. The filtrate was reacted with ferric chloride solution (3–4 drops), and the formation of precipitate was observed. The appearance of bluish black colored precipitate indicated the presence of phenol.

#### 2.4.5. Test for Glycosides

The presence of glycosides in the PO husk was evaluated by the Keller-Kiliani test [2]. Extract (2 mL) was reacted with glacial acetic acid (1 mL) and FeCl_3_ (1–2 drops), then treated with concentrated H_2_SO_4_ (1 mL). Development of green blue color indicated the presence of cardiac glycosides and vice versa.

### 2.5. Preparation of Orodispersible Tablets Using PO Husk as Disintegrant

A direct compression process was used for the preparation of ODTs using domperidone as the model drug. Various formulations of ODTs were developed using different concentrations of the PO husk as disintegrant as shown in Table 1. All the ingredients were weighed, sifted, and mixed for 15 min in a lab scale double cone blender at 25 rpm. This powder mixture was compressed by a ZP-19 rotary compression machine (STC, China) with 10 mm round, shallow concave punch fitting. A minimum of 500 tablets were prepared for all formulations.

### 2.6. Precompression Evaluation

Prior to compression, the powder blend of all the formulations was evaluated for various parameters related to flow-like bulk density, tapped density, Carr's index, Hausner ratio, angle of repose, and interparticle porosity, as per Section 2.3.

### 2.7. Postcompression Evaluation

Compressed ODTs were extensively evaluated for various official and unofficial parameters, as follows.

#### 2.7.1. Physical Parameters of Tablets

Experimental tablet weight variation from all formulations was determined by weighing 20 tablets individually [20] on a digital balance (Precisa, Switzerland). While the thickness of ten randomly chosen tablets was tested by a digital tester (Pharma Test, Germany), the mean and standard deviation were calculated. Domperidone quantity of the ODTs was estimated as per the official British Pharmacopoeia method [18]. Absorbance of the test and standard solutions was noted at 384 nm using a double beam UV-visible spectrophotometer (PerkinElmer, USA), and drug percentage was determined. A reported method was used to calculate tablet wetting time using filter paper [21]. A filter paper folded twice was taken in a Petri dish having 5 mL of purified water; the tablet was then put on the paper and the time for thorough wetting was noted.

#### 2.7.2. Mechanical Properties of ODTs

The mechanical strength of ODTs was measured based on tablet crushing strength, specific crushing strength, tensile strength, and friability. For every formulation, the crushing strength was calculated on ten randomly chosen tablets (*n* = 10), by digital hardness and thickness tester (Pharma Test, Germany). The mean crushing strength and thickness values were then utilized in the estimation of tablet tensile strength and specific crushing [18] using equations (7) and (8), respectively, viz., *T* = 2*F*/*πDH* (equation (7)) and *τ* = *F*/*HD* (equation (8)), where *T* is the tensile strength of tablets (N/mm^2^), *τ* is the specific hardness of the tablet (N/mm^2^), *F* is the crushing strength of the tablet (N), *H* is the thickness of the tablet (mm), *D* is the diameter of the tablet (mm), and *τ* is the proportionality constant having value of 3.143. The formulation tablet friability was estimated in accordance with official compendia [18], by a single drum friabilator (Faisal Engineering, Pakistan).

#### 2.7.3. Disintegration Behavior of ODTs

Disintegration behavior of ODTs was estimated on the basis of disintegration time and oral disintegration time. Tablet disintegration time (D.T) was carried out in distilled water at 37 ± 2°C as per official USP [17]. The mean of the disintegration time of six tablets was taken as the disintegration time of the tablet.

Oral D.T of ODTs was assessed by a panel of six healthy male volunteers. Prior to the test, every subject was asked to rinse his oral cavity with purified water (200 mL). One tablet was placed on the tongue of the volunteer, and a stopwatch was started directly. All subjects were taught to cause a tumbling action by moving the tablet slowly against the upper portion of the oral cavity and evading side-to-side tumbling or biting. The time taken for complete disintegration of the tablet was noted. Mean of six determinations (*n* = 6) was taken as the oral disintegration time. At the end of the test, each volunteer was asked about the grittiness felt in the oral cavity and to rank the taste of the tablet as per the following scale: 0: tasteless, 1: pleasant tasting, 2: sweet, and 3: strongly sweet. As human volunteers were involved in the determination of the oral disintegration time, the study was permitted by the Committee for Ethics in Research at the Department of Pharmacy, Abasyn University Peshawar, and written consent forms were signed by all the participants.

#### 2.7.4. In Vitro Drug Release

The dissolution rate of domperidone was estimated as per the official British Pharmacopoeia [20] with a dissolution medium which used 0.1 N hydrochloric acid (900 mL) held at 37 ± 2°C in a USP Dissolution Apparatus-ІІ (paddle method). The operation speed of the paddle was set at 50 rpm [20]. At defined time intervals, a sample (5 mL) was taken and fresh medium was added (0, 5 10, 15, 30, 45, and 60 min) and filtered. Total drug release was estimated by measuring the UV absorbance of the sample at 284 nm using a double beam UV spectrophotometer (Shimadzu, Japa3).

## 3. Results

**Figure 1:**
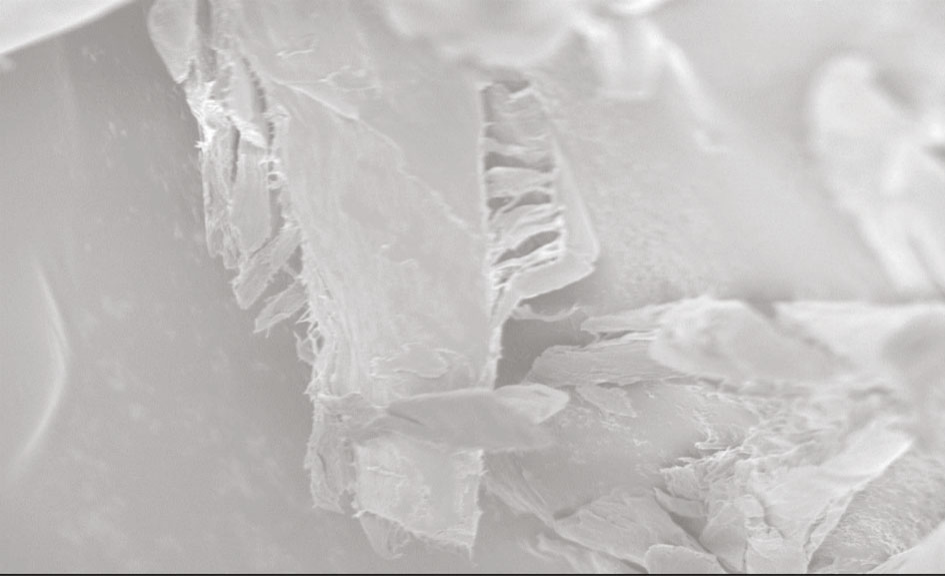
SEM photograph of PO husk fibers.

**Figure 2:**
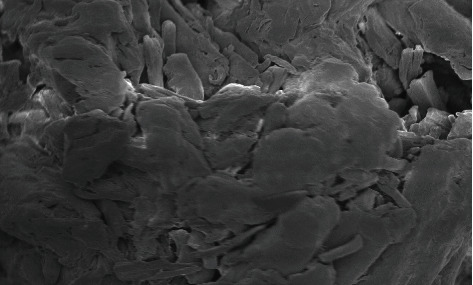
SEM photograph showing closer look at the surface of PO husk.

**Figure 3:**
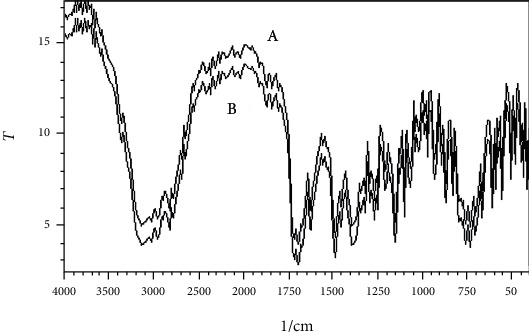
IR spectra of domperidone (A) and mixture of domperidone, PO husk, and other excipients after subjecting to stress conditions (40 ± 2°C and 75 ± 5% relative humidity) for 15 days.

**Figure 4:**
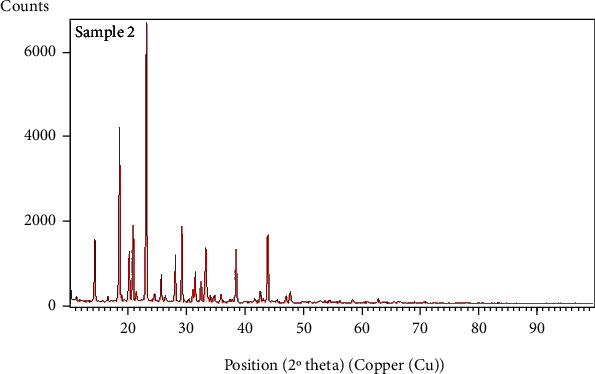
Results of XRD analysis of mixture of PO husk with other ingredients used in formulation of ODTs.

The swelling index of the PO husk was very high (380%) indicating its good water absorbing capacity. Pulverization of the husk into fine powder has an adverse effect on the swelling index. The swelling index of the powdered PO husk was 310%, which was very low as compared with unpulverized. The PO husk has a low density with poor compressibility, as shown in Table 2. The PO husk was unable to flow, and its angle of repose cannot be determined. Other parameters related to flow also showed poor results. Phytochemical evaluation of the PO husk was performed according to the standard protocols, and the presence of different phytochemicals was confirmed, as shown in Table 3. Prior to compression, the powder blend was evaluated for various parameters like bulk density, tapped density, Hausner ratio, Carr's index, and angle of repose, according to the official method (USP) [18]. The powder blend for all the formulations had better flow as evidenced from the results presented in Table 4.  Figure 1 shows a SEM photograph of the PO husk, indicating its fibrous nature. A closer look of the surface showed that it was very rough, providing a larger surface area for water absorption, as shown in Figure 2.  Figure 3 shows the IR spectra of pure domperidone and the spectra of a physical mixture of the PO husk, domperidone, and other ingredients (as per Table 1) in 1 : 1 by weight, after subjecting to stress conditions (40 ± 2°C and 75 ± 5% relative humidity) for 15 days.  Figure 4 shows the results of the XRD analysis of a mixture of PO husk and excipients used in the formulation of ODTs. The wetting time for all the formulations was lower than that of the control formulation (F-1) which did not contain the PO husk. Increase in the concentration of the PO husk resulted in decreased wetting, as shown in Table 5. A smaller wetting time shows good absorption and distribution of water by ODTs, which was because of the hydrophilic nature of the PO husk. A direct correlation was observed between the quantity of the husk and the decrease in the wetting time, i.e., the increase in quantity of the PO husk decreased the wetting time.

ODTs disintegrate in the oral cavity, and all the ingredients (API+excipients) are in direct contact with oral mucosa and taste buds. It makes evaluation of the mouth feel with ODTs a mandatory test. Bad taste and grittiness are commonly observed upon oral ingestion, and the bad (usually bitter) taste is due to API while grittiness is caused by the excipients. Domperidone is a tasteless drug, and the taste of ODTs further improved by inclusion of a sweetener (aspartame 3% *w*/*w*) and flavor (0.5% *w*/*w*). The PO husk was expected to cause grittiness due to its high water absorbing capacity. Upon contact with saliva, it forms a thick gel and sticks to the oral mucosa, creating a very bad sensation. So, placebo (drug free) ODTs containing different concentrations of the PO husk (within the range of 0.5–10% *w*/*w*) were prepared and evaluated by a panel of healthy human volunteers (*n* = 6) for taste and mouth feel. Grittiness of the tablets was ranked from 0 to 3, with 0 as no grittiness. The highest quantity of the PO husk in placebo ODTs was 10% *w*/*w*. It caused no grittiness at this level as shown in Table 6. Some grittiness was observed at the 10% concentration as two volunteers reported mild grittiness. The rest of the volunteers declared the tablet free of grittiness. Mechanical strength of ODTs was assessed on the basis of their crushing strength, specific crushing strength, tensile strength, and friability, determined according to official compendia [20]. All the parameters were determined in accordance with USP. The crushing strength of ODTs was in the range of 3.67–5.59 kg, indicating good crushing strength. Similarly, results of other parameters like tensile strength, specific crushing strength, and friability were observed, indicating good mechanical strength. Moreover, friability of all the formulations was within the acceptable range (not more than 0.8%). It may be due to the fibrous nature of the PO husk which created bonding among the ingredients of ODTs (Table 7).

The disintegration time and oral disintegration time are the two major indicators for determining the disintegration behavior of ODTs. In the present study, the disintegration time was determined according to USP, using purified water as disintegration media at 37 ± 2°C. All the tablets had disintegration time within the official limits for ODTs (not more than 3 min). The lowest disintegration time was observed with formulation F-6, containing 10% PO husk. The PO husk rapidly absorbs water and swells to a large extent, causing tablet disintegration. Oral disintegration time was determined by a panel of healthy male volunteers (*n* = 6). The British Pharmacopoeia [18] recommends 0.1 N HCl (900 mL) as dissolution media, stirred with apparatus-II (peddle method) at 50 rpm, for dissolution testing of conventional tablets of domperidone. The dissolution rate of ODTs was determined using the same dissolution media and under the same experimental conditions. Complete (100%) drug release was observed with all the formulations, irrespective of the amount of disintegrant. Furthermore, the strength of ODTs was 10 mg/tablet, and it has good solubility in 0.1 N HCl, so the dose-to-solubility ratio was high and the sink condition developed during dissolution testing.

## 4. Discussion

In the present study, the husk of PO seeds was evaluated for its suitability to be used as a pharmaceutical excipient for solid dosage form (tablet). The PO husk was evaluated for various pharmaceutical parameters like density, Hausner ratio, Carr's Index, angle of repose, solubility, loss on drying, and compressibility. Results showed that the PO husk was unable to flow, but it increased the flow of the powder blend significantly. The PO husk had a high swelling index, resulting in rapid disintegration of ODTs in a concentration-dependent manner. Furthermore, it had a positive effect on the mechanical strength of ODTs. The PO husk is water insoluble, but it absorbs a large quantity of water and swells up. To get better water absorption and subsequent swelling and disintegration, the PO husk should be used without pulverization. It has been reported that the PO husk mainly contains carbohydrates and glycosides [20], and results presented in Table 3 are in agreement with the reported literature. The direct compression technique was applied for the preparation of ODTs of domperidone, using the PO husk as a disintegrant in different concentrations. The PO husk has poor flow and compressibility, and its adverse effect on the powder blend was expected. Better compressibility and flow characteristics of Tablettose-80 compensated for the poor flow of the PO husk. Furthermore, all the formulations containing the PO husk showed better flow than the control formulation (F-1) which may be because of the glidant effect of the husk due to its bulky and slippery fibrous nature.

The PO husk was found compatible with domperidone and other ingredients, used in the formulation of ODTs. All the characteristic peaks were present in both the spectra, and no shift was observed, indicating compatibility of the ingredients with each other. Compatibility of the PO husk with other ingredients of the formulation was further confirmed by its XRD analysis, as shown in Figure 4. ODTs of domperidone were prepared with 10 mm round, shallow concave punches. Compression weight of the tablets was 150 mg, and weight variation was in a narrow range, as shown in Table 5. Results of the weight variation showed that the flow of the powder blend was not affected by the PO husk. Physically, tablets from all the formulations had a smooth and shiny surface and were free of sticking and picking, indicating proper lubrication. Results showed that ODTs containing the PO husk as disintegrant will be free of grittiness. In concentrations below 10%, the PO husk forms a paste-like mass upon water absorption which was smooth and devoid of grittiness [5].

**Figure 5:**
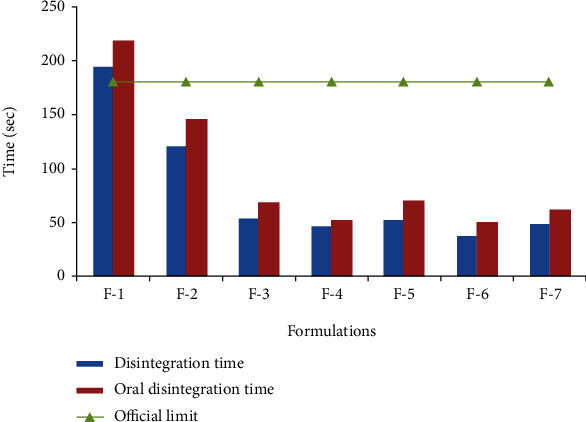
Disintegration time and oral disintegration time of ODTs containing PO husk.

**Figure 6:**
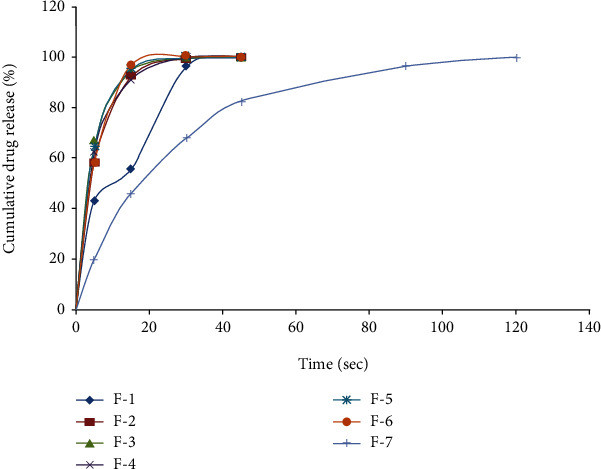
*In vitro* drug release of domperidone from ODTs containing PO husk as disintegrant. Dissolution rate was determined in 0.1 N HCl (900 mL), using USP apparatus-II (peddle) at 50 rpm.

The PO husk is bulky in nature and has poor compressibility, and ODTs were expected to be soft. But ODTs had good mechanical strength, and poor compressibility of the PO husk was compensated by other excipients. The highest concentration of *the* PO husk (15% *w*/*w*) was in formulation F-7, and it had a good mechanical strength which proved that it had no adverse effect on the mechanical strength of ODTs. All the formulations showed rapid disintegration in the oral cavity. A direct correlation was observed between the *in vitro* disintegration time and the oral disintegration time, as shown in Figure 5. Decrease in the disintegration time was accompanied by a decrease in the oral disintegration time and vice versa. The PO husk showed a concentration-dependent decrease in the disintegration time. Furthermore, addition of other disintegrants resulted in a synergistic effect. The main disadvantage of available synthetic disintegrants is the low mechanical strength of tablets. It has been reported that use of a large quantity of super disintegrants results in tablets with poor friability. Poor friability is more pronounced in the case of ODTs which are compressed at a relatively lower compression force. The PO husk overcomes the problem by increasing the mechanical strength through the formation of a strong linkage with other particles which strengthens the ODTs. Furthermore, it absorbs water rapidly due to its high swelling index, resulting in rapid disintegration. All the formulations containing the PO husk up to 10% (F-2 to F-5) showed similar drug release profiles, and differences were made on the basis of burst release, i.e., drug release during the initial 15 minutes. The PO husk showed a concentration-dependent burst release of the drug and an increase in the concentration of the PO husk resulted in a higher drug release. Drug release from ODTs can be correlated with their disintegration time. All the formulations with a smaller disintegration time showed a higher burst release, as shown in Figure 6. It may be due to the quick exposure of the drug to dissolution media upon rapid disintegration which resulted in better dissolution. Rapid disintegration and sink condition are responsible for burst release from ODTs. Formulation F-1 was used as a control and was devoid of the PO husk and other disintegrants. It showed the largest disintegration time (194 sec) and the lowest drug release (75.81 ± 1.26%) during the initial 15 min. Inclusion of a higher concentration of PO husk (15% *w*/*w*) resulted in a slower drug release which may be due to its gelling action. At a higher concentration, the PO husk forms a gel-like structure which decreased the drug release. However, the resultant gel was not strong enough to retard the drug release for a longer period of time. So the PO husk can be used as a pharmaceutical excipient (disintegrant and glidant), without compromising the mechanical strength of tablets.

## 5. Conclusions

It was concluded that the PO husk could be used as a disintegrant in the formulation of ODTs.

## Figures and Tables

**Table 1:** Composition of various formulations of orodispersible tablets of domperidone containing PO husk as disintegrant, prepared by direct compression technique.

Ingredients	F-1 (control)	F-2	F-3	F-4	F-5	F-6	F-7
Domperidone	6.67	6.67	6.67	6.67	6.67	6.67	6.67
Flavor (orange)	0.50	0.50	0.50	0.50	0.50	0.50	0.50
Aspartame (sweetener)	3.00	3.00	3.00	3.00	3.00	3.00	3.00
Primojel	3.00	—	—	—	2.50	—	—
*Plantago ovata* husk	—	3.00	5.00	7.50	2.50	10.00	15.00
Microcrystalline cellulose (Ph102)	50.00	50.00	50.00	50.00	50.00	50.00	50.00
Tablettose-80	35.33	35.33	33.33	30.83	33.33	28.33	23.33
Magnesium stearate	1.50	1.50	1.50	1.50	1.50	1.50	1.50

Note: quantities are given in % (*w*/*w*).

**Table 2:** Pharmaceutical characterization of PO husk.

Parameter (unit)	Result
Bulk density (g/mL)	0.249 ± 0.004 (*n* = 3)
Tapped density (g/mL)	0.282 ± 0.001 (*n* = 3)
Carr's index	12.41
Hausner ratio	1.13
Angle of repose (°)	0.00
Flowability (sec)	0.00
Swelling index (unprocessed) (%)	380 ± 5.29 (*n* = 3)
Swelling index (powdered) (%)	310 ± 3.64 (*n* = 3)
Interparticle porosity	0.499
Loss on drying (%)	1.87 ± 0.13 (*n* = 3)
Compressibility	0.00

Note: results are presented as mean ± SD (*n* = 3); time taken by 100 g husk to flow through an orifice; parameter has no unit because it is the ratio between two parameters with same units; powder was unable to flow and hence the parameter was unable to be determined.

**Table 3:** Phytochemical constituents of PO husk.

Test for presence of	Observations	Result
Alkaloids	Colored precipitate present with these reagents	Alkaloids present
Tannins	Dark green solution/blue-black precipitate	Tannins present
Saponins	Persistent froth	Saponins present
Flavonoids	Yellow solution that turns to colorless	Flavonoids present
Glycosides	Reddish brown layer formed at interface	Glycosides present
Phenols	Formation of green precipitates indicate phenols	Phenols present

**Table 4:** Precompression evaluation of powder blend containing PO husk in different concentrations.

Characteristics (unit)	F-1 (control)	F-2	F-3	F-4	F-5	F-6	F-7
Bulk volume (mL)	25.00	25.00	25.00	25.00	25.00	25.00	25.00
Tapped volume (mL)	23.00	22.00	23.50	23.00	23.00	20.00	18.00
Bulk density (g/mL)	0.48	0.44	0.43	0.47	0.48	0.39	0.38
Tapped density (g/mL)	0.52	0.47	0.45	0.51	0.50	0.41	0.40
Hausner ratio^∗^	1.11	1.06	1.06	1.09	1.05	1.05	1.07
Carr's index^∗^	7.85	5.77	5.74	8.06	4.59	4.62	6.45
Angle of repose (°)	36.16	27.95	25.42	30.61	30.72	23.02	22.01

^∗^The given parameters have no unit as these are ratios between parameters with same units.

**Table 5:** Physical parameters of ODTs of domperidone prepared by direct compression technique.

Formulation code	Weight variation (%)	Tablet thickness (mm)	Tablet diameter (mm)	Wetting time (sec)	Drug content (%)
F-1 (control)	±2.19	3.91 ± 0.81	7.46 ± 0.08	185.66 ± 1.624	100.08 ± 0.73
F-2	±1.09	3.9 ± 0.28	7.45 ± 0.04	71.33 ± 1.307	99.86 ± 0.49
F-3	±2.21	3.98 ± 0.52	7.48 ± 0.07	59.66 ± 1.753	99.94 ± 1.19
F-4	±3.30	3.88 ± 0.92	7.51 ± 0.02	42.33 ± 1.945	100.24 ± 0.28
F-5	±2.23	3.92 ± 0.40	7.52 ± 0.06	58.33 ± 1.292	100.01 ± 0.93
F-6	±3.67	3.90 ± 0.64	7.45 ± 0.01	59.66 ± 1.922	99.68 ± 0.71
F-7	±2.38	3.92 ± 0.61	7.58 ± 0.02	42.30 ± 1.809	99.81 ± 0.79

**Table 6:** Level of grittiness caused by placebo (drug-free) ODTs containing different concentrations of PO husk.

Quantity of PO husk (% *w*/*w*)	Quantity of sweetener+flavor (% *w*/*w*)	Number of volunteers rated ODTs as			
		0	1	2	3
1.00	3.00 + 0.50	6	—	—	—
2.50	3.00 + 0.50	6	—	—	—
5.00	3.00 + 0.50	6	—	—	—
7.50	3.00 + 0.50	—	5	1	—
10.00	3.00 + 0.50	—	2	4	—

0: no grittiness; 1: acceptable; 2: mild grittiness; 3: grittiness.

**Table 7:** Mechanical strength of ODTs containing different concentrations of PO husk, prepared by direct compression.

Formulation code	Crushing strength (kg)	Tensile strength (kg/mm^2^)	Specific crushing strength (kg/mm^2^)	Friability (%)
F-1	3.67 ± 0.43	0.080	0.125	0.74
F-2	3.73 ± 0.93	0.081	0.128	0.24
F-3	4.24 ± 0.71	0.090	0.142	0.71
F-4	4.19 ± 0.58	0.087	0.137	0.72
F-5	4.41 ± 0.09	0.095	0.149	0.24
F-6	5.51 ± 0.36	0.120	0.189	0.49
F-7	5.48 ± 0.68	0.117	0.184	0.24

## Data Availability

Upon request data could be requested from the shared corresponding author Dr. Amjad Khan dr.amjad@kust.edu.pk.
